# Effect of viscosity of experimental universal adhesive on bond strength to dentin prepared with Er:YAG laser

**DOI:** 10.1038/s41598-023-34984-1

**Published:** 2023-05-16

**Authors:** Takehiro Kamitsu, Junko Shimomura-Kuroki, Koichi Shinkai

**Affiliations:** 1 Advanced Operative Dentistry-Endodontics, The Nippon Dental University Graduate School of Life Dentistry at Niigata, 1-8 Hamaura-cho, Chuo-ku, Niigata, 951-8580 Japan; 2 Department of Pediatric Dentistry, The Nippon Dental University School of Life Dentistry at Niigata, 1-8 Hamaura-cho, Chuo-ku, Niigata, 951-8580 Japan; 3 Department of Operative Dentistry, The Nippon Dental University School of Life Dentistry at Niigata, 1-8 Hamaura-cho, Chuo-ku, Niigata, 951-8580 Japan

**Keywords:** Dental biomaterials, Bonded restorations

## Abstract

The aim of this study was to clarify the effect of universal adhesive (UA) viscosity on the bond strength of resin composite to dentin prepared with Er:YAG laser. Four experimental UAs (SI-1, SI-2, SI-3, and SI-4) were developed by adding 1, 2, 3, and 4 wt/% nanosilica to BeautyBond Xtreme (Shofu), respectively. BeautyBond Xtreme was used as a control (SI-0). The viscosities of experimental UAs were measured using a B-type viscometer. After bovine mandibular anterior teeth were ground with #600 emery paper to obtain the flattened dentin surfaces, the dentin surfaces were cut thinly by irradiating the Er:YAG laser. Specimens were prepared using the respective UA and flowable resin composite and subjected to the microtensile bond strength (µTBS) test. The data from viscosity measurement and the μTBS test were statistically analyzed using the Kruskal–Wallis test. The mean values of viscosity significantly differed among the all experimental groups (*p* < 0.01). The μTBS of SI-1 and SI-2 was significantly higher than that of SI-0, SI-3, and SI-4 (*p* < 0.001). The μTBS of SI-0 was significantly lower than that of SI-4 (*p* < 0.001). The viscosities of the experimental universal adhesives significantly affected their bond strength to laser-cut dentin.

## Introduction

Conventionally, the removal of dental caries has been mostly achieved using rotary cutting instruments. However, the Er:YAG laser has recently been used for removing dental caries in clinics because its ability to cut hard tissues was improved. Caries removal devices include rotary cutting apparatus (air-turbine and micromotor), ultrasonic scalers^1^, air abrasion^2^, manual cutting instruments with chemical agent^3^, and dental lasers such as the Er:YAG laser^4,5^. Among these devices, the Er:YAG laser possesses the advantage of no unpleasant noise, vibration, and pain during tooth-cutting^4^. Cavity preparation with the Er:YAG laser could be useful for nervous patients including Children because they may refuse cavity preparation using rotary cutting apparatus, which accompanies noise and tooth vibration during tooth cutting due to their feeling of discomfort, anxiety, and fear of noise and tooth vibration^5^.

Previously, many studies have investigated dentin surfaces irradiated with lasers^6–10^, and it has been reported that the surface of laser-irradiated dentin was devoid of a smear layer and had a complex mica-like shape with opened dentin tubules^9,10^. Previous studies have revealed the microscopic morphology of laser-etched enamel and have shown that enamel surfaces irradiated with Er:YAG lasers were as rough as enamel surfaces treated with phosphates^11–13^.

Micro-morphological changes on tooth surfaces caused by laser irradiation affect the bond strength to resin composite (RC). Kameyama et al. have reported that the bond strength of RC to the laser-irradiated enamel was equivalent to that of the phosphate-treated enamel^11^. Meanwhile, other studies have reported that the bond strength of RC to the laser-irradiated enamel was lower than that to the phosphate-treated enamel^12,13^. Although a few studies reported that laser irradiation showed no effect^14,15^ or positive effect^16,17^ on the dentin bond strength of RC, many studies reported that the bond strength of RC to laser-irradiated dentin was low because adhesive monomer infiltration was disturbed by the heat-denatured layer generated with laser irradiation^12,18–22^.

Previous studies investigated treatments to improve the bond strength of RC to laser-irradiated dentin and reported various useful treatments: removing the laser-irradiated dentin surface using various instruments^18,23^, modifying the laser-irradiated dentin surface by re-irradiating laser under different conditions^24^, modifying the laser-irradiated dentin surface using various chemical agents^18,21,23,25,26^, and applying the universal adhesive with rubbing^27^. However, these treatments did not make the bond strength of RC to the laser-irradiated dentin recover to equivalent to that of the rotary-cut dentin, moreover, possess the disadvantage of more operating steps^24–26^.

Recently, advances in adhesive systems have simplified self-etching adhesive systems from two-step to one-step and shortened the application time of self-etching adhesive; thus, the operation time required for tooth surface treatment has been reduced^28^. Universal adhesive (UA) in one-step adhesive systems, which can bond to various adherends such as ceramics, metals, and dental substances, are now being used in clinics. In pediatric dentistry, longer treatment worsens the cooperation of younger patients for dental treatment. Hence, UA could be useful for RC restoration in pediatric caries treatment due to a short application time^15^. Thus, RC restorations using a combination of laser cutting and tooth surface treatment with UA appear to be very effective in the treatment of dental caries in children^29–31^.

A previous study which investigated the bond strength of RC to Er:YAG laser-irradiated dentin using several types of commercially available universal adhesives (UAs) revealed that the adhesive monomer in the lower viscosity bonding agent infiltrated into the healthy dentin beneath the laser-affected dentin; thus, the lower viscosity UA showed higher bond strength of RC to laser-irradiated dentin than the higher one^32^. However, the proper effect of the viscosity of bonding agents on the bond strength of RC to the laser-irradiated dentin may be unclear because the bonding agents used in previous studies were from different manufacturers; thus, other factors such as mechanical retention yielded resin tags might affect the bond strength. Therefore, we experimentally developed UA with different viscosities by adding nanosilica to a resin matrix of the same composition. This study aims to clarify the effect of the viscosity of experimentally developed UAs on the bond strength of RC to Er:YAG laser-cut bovine dentin. The null hypothesis is that the viscosity of experimentally developed UAs would not influence the bond strength of RC to Er:YAG laser-cut bovine dentin.

## Results

The data obtained from viscosity measurement in each group were not normally distributed except SI-0. The data obtained from μTBS test in each group were not normally distributed except SI-4 in both L and G group. The data obtained from both viscosity measurement and μTBS test did not show homogeneity of variance. Accordingly, both data obtained from viscosity measurement and μTBS test were statistically analyzed using the Kruskal–Wallis and Steel–Dwass tests. In addition, a significant difference between the L and G groups was examined using Mann–Whitney's U test. The adhesive specimens in L group were made from ground, flattened dentin surfaces with laser irradiation, while the adhesive specimens in G group were made from flattened dentin that has only been ground.

### Viscosity measurement

**Figure 1:**
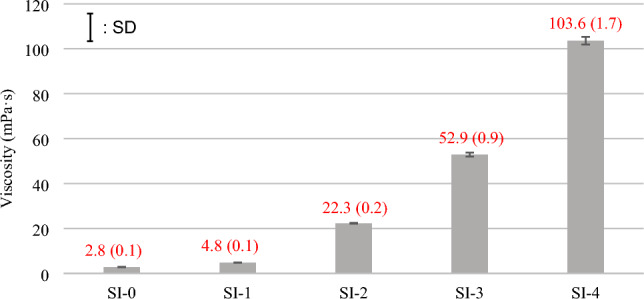
Viscosity of experimental universal adhesives.

The added amount of nanosilica changed the experimental UA viscosity significantly, and the viscosity was increased doubling with the amount of nanosilica added between SI-0 and SI-1, SI-2 and SI-3, and SI-3 and SI-4. The results of viscosity measurement (mean and SD) are shown in Fig. 1. There were significant differences among the all-experimental groups (*p* < 0.01).

### μTBS measurement

**Figure 2:**
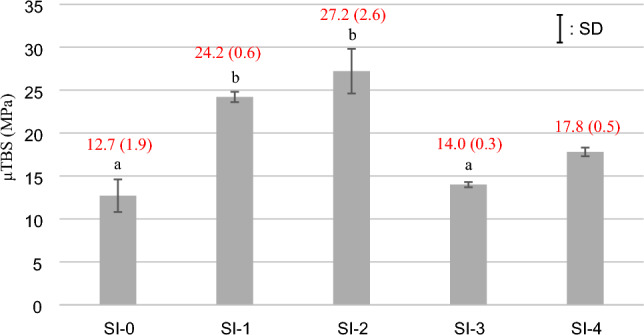
The results of μTBS measurement on the L group (mean and SD). The same lowercase letters indicate no significant difference (*p* > 0.05) among the experimental groups in the L group.

**Figure 3:**
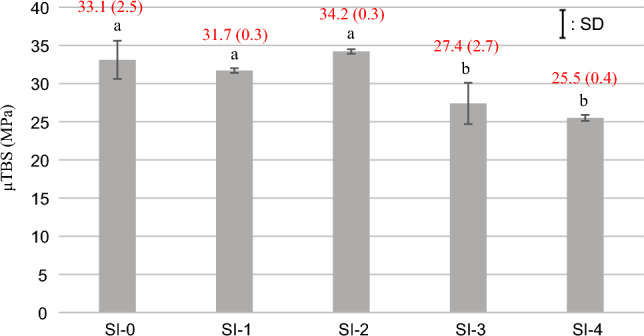
The results of μTBS measurement on the G group (mean and SD). The same lowercase letters indicate no significant difference (*p* > 0.05) among the experimental groups in the G group.

The results of μTBS measurement (mean and SD) in the L and G group are shown in Figs. 2 and 3, respectively.

In the L group, each μTBS of SI-1 and SI-2 was significantly higher than that of SI-0, SI-3, and SI-4 (*p* < 0.001). There was no significant difference in μTBS between SI-1 and SI-2 as well as SI-0 and SI-3. The μTBS of SI-0 was significantly lower than that of SI-4 (*p* < 0.001).

In the G group, each μTBS of SI-0, SI-1, and SI-2 was significantly higher than that of SI-3 and SI-4 (*p* < 0.001). No significant difference in μTBS was detected between SI-0 and SI-1, SI-0 and SI-2, SI-1 and SI-2, and SI-3 and SI-4. The comparison between the L and G groups in the same experimental UA showed that the µTBS of the G group was significantly higher than that of the L group (*p* < 0.001).

### Failure mode analysis

**Figure 4:**
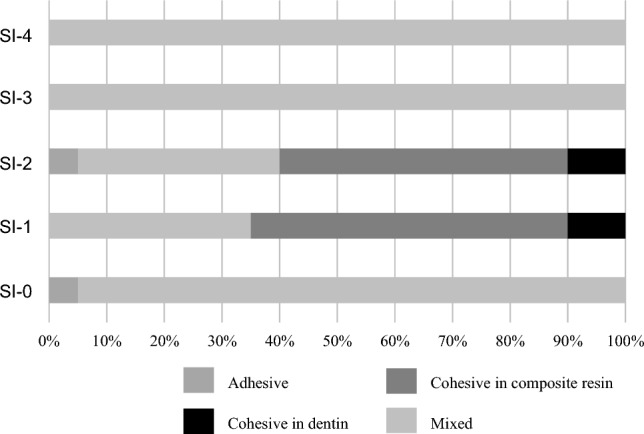
The result of failure mode analysis on the L group.

In the L group, the specimens shown higher bond strength, such as treated with SI-1 and SI-2, tended to show a higher ratio of cohesive failure in RC, whereas the specimens shown lower bond strength, such as treated with SI-0, SI-3, and SI-4, showed almost mixed failure (Fig. 4).

**Figure 5:**
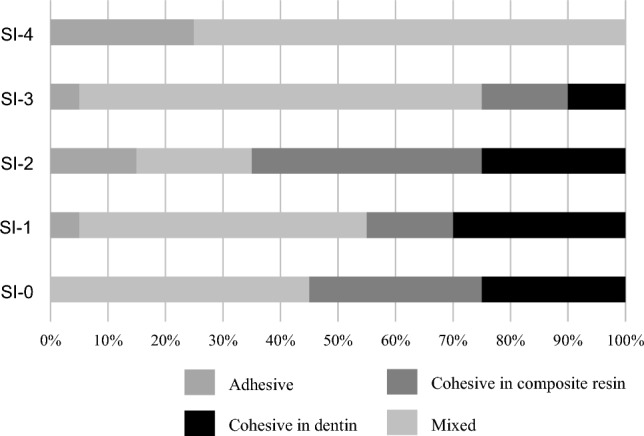
The result of failure mode analysis on the G group.

In the G group, the specimens shown higher bond strength, such as treated with SI-0, SI-1, and SI-2, tended to show a higher ratio of cohesive failure, whereas the specimens shown lower bond strength, such as treated with SI-3 and SI-4, tended to show a higher ratio of mixed failure (Fig. 5).

### SEM observation

**Figure 6:**
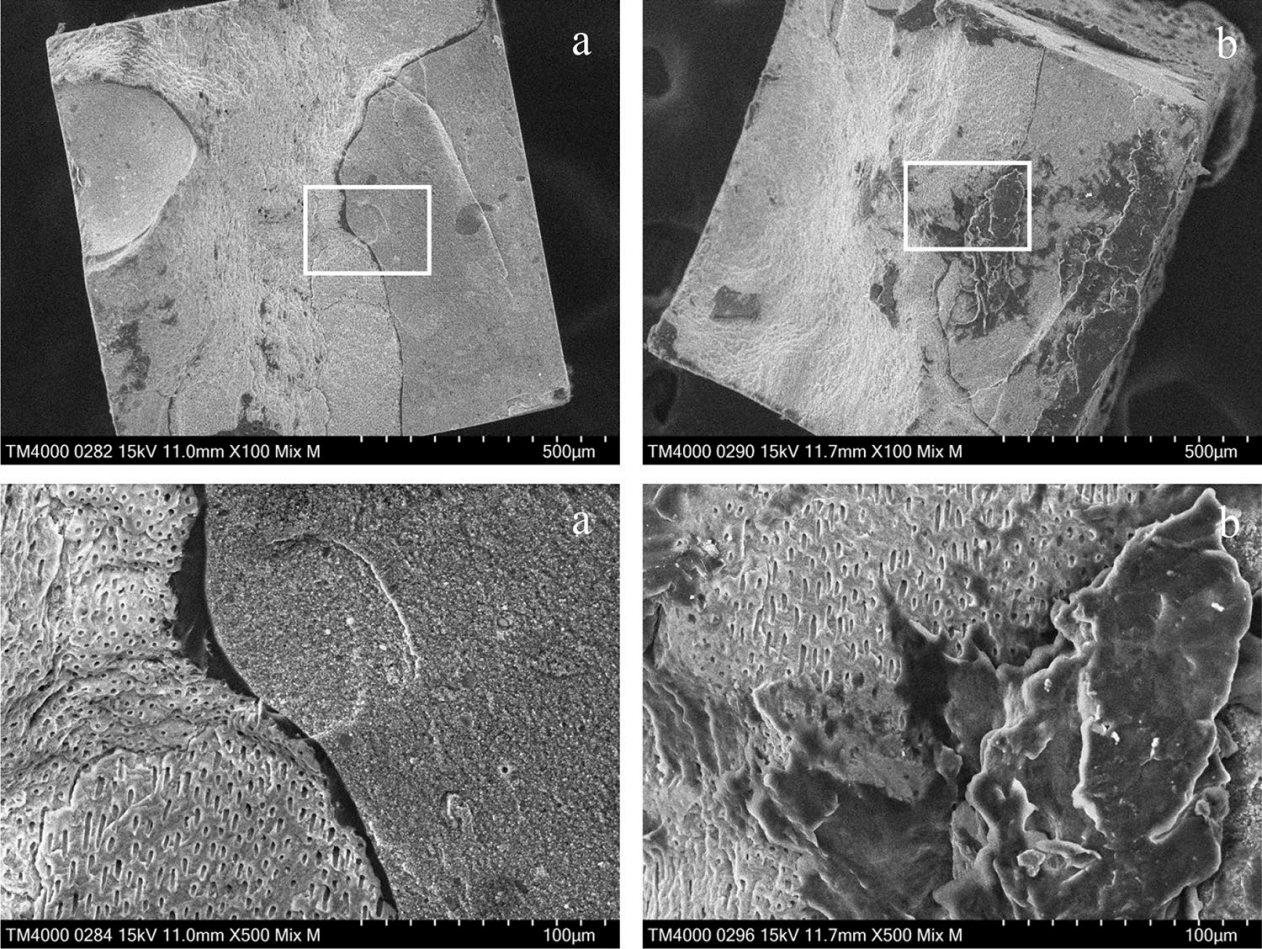
SEM images of the fractured surfaces of the specimen treated with SI-0 in L group. (**a**) RC site, (**b**) dentin site. Magnification: upper × 100, lower × 500. A dentin fracture fragment is observed on the beam resin site, and a scale-like dentin fracture surface and small fracture resin fragment are observed on the beam dentin site. The dentin tubules of fragments are open.

**Figure 7:**
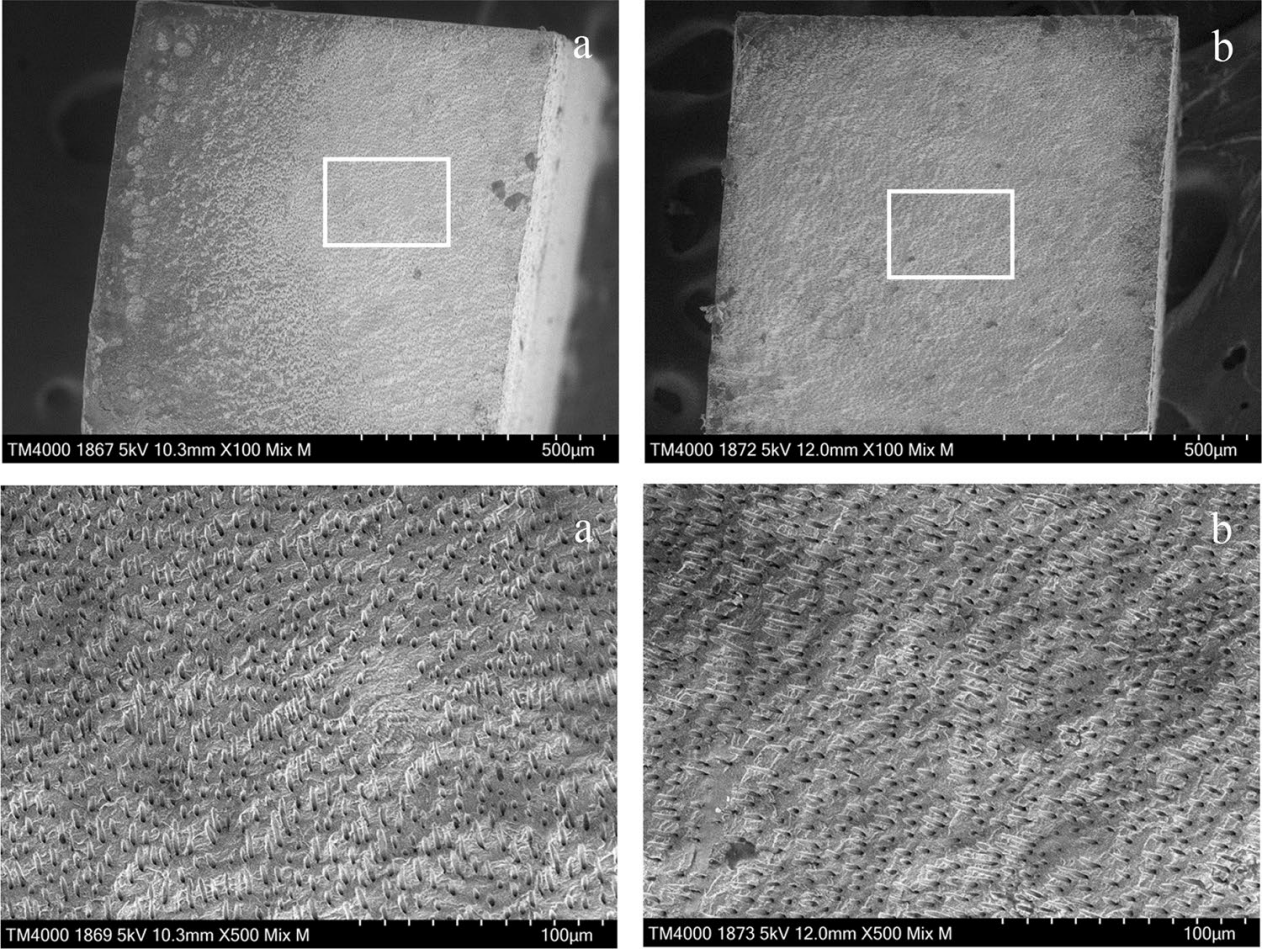
SEM images of the fractured surfaces of the specimen treated with SI-2 in L group. (**a**) RC site, (**b**) dentin site. Magnification: upper × 100, lower × 500. The dentin fracture surfaces are observed on both the beam RC and dentin sites. The dentin tubules are open.

SEM images of the fractured surfaces of representative specimens in the L group are shown in Figs. 6 and 7. Figure 6 shows the fracture surface of the specimen treated with SI-0, which was judged as mixed failure. In Fig. 6, a dentin fracture fragment is observed on the RC site of the beam, and a scale-like dentin fracture surface and small fracture RC fragment are observed on the beam dentin site. Figure 7 shows the fracture surface of the specimen treated with SI-2, which was judged as a cohesive failure in dentin. In Fig. 7, the fracture dentin surfaces are observed on both the beam resin and dentin sites.

**Figure 8:**
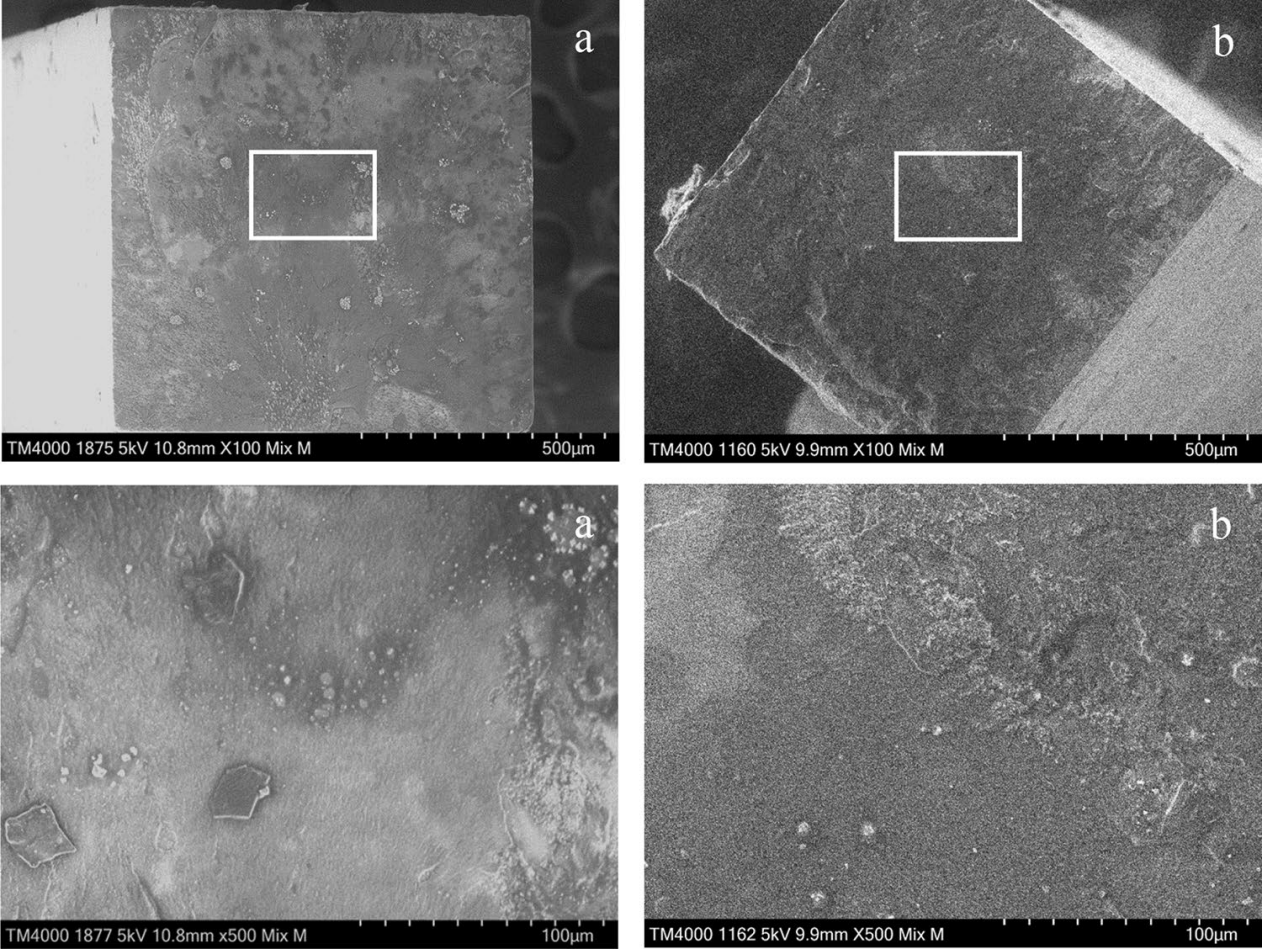
SEM images of the fractured surfaces of the specimen treated with SI-2 in G group. (**a**) RC site, (**b**) dentin site. Magnification: upper × 100, lower × 500. A thin universal adhesive layer is observed on the dentin site.

SEM image of the fractured surface of the G group is shown in Fig. 8. Figure 8 shows the fracture surface of the specimen treated with SI-2, which was judged as an adhesive failure. In Fig. 8, a small fragment of fractured dentin is observed on the RC site, and a thin UA layer is observed on the dentin site.

**Figure 9:**
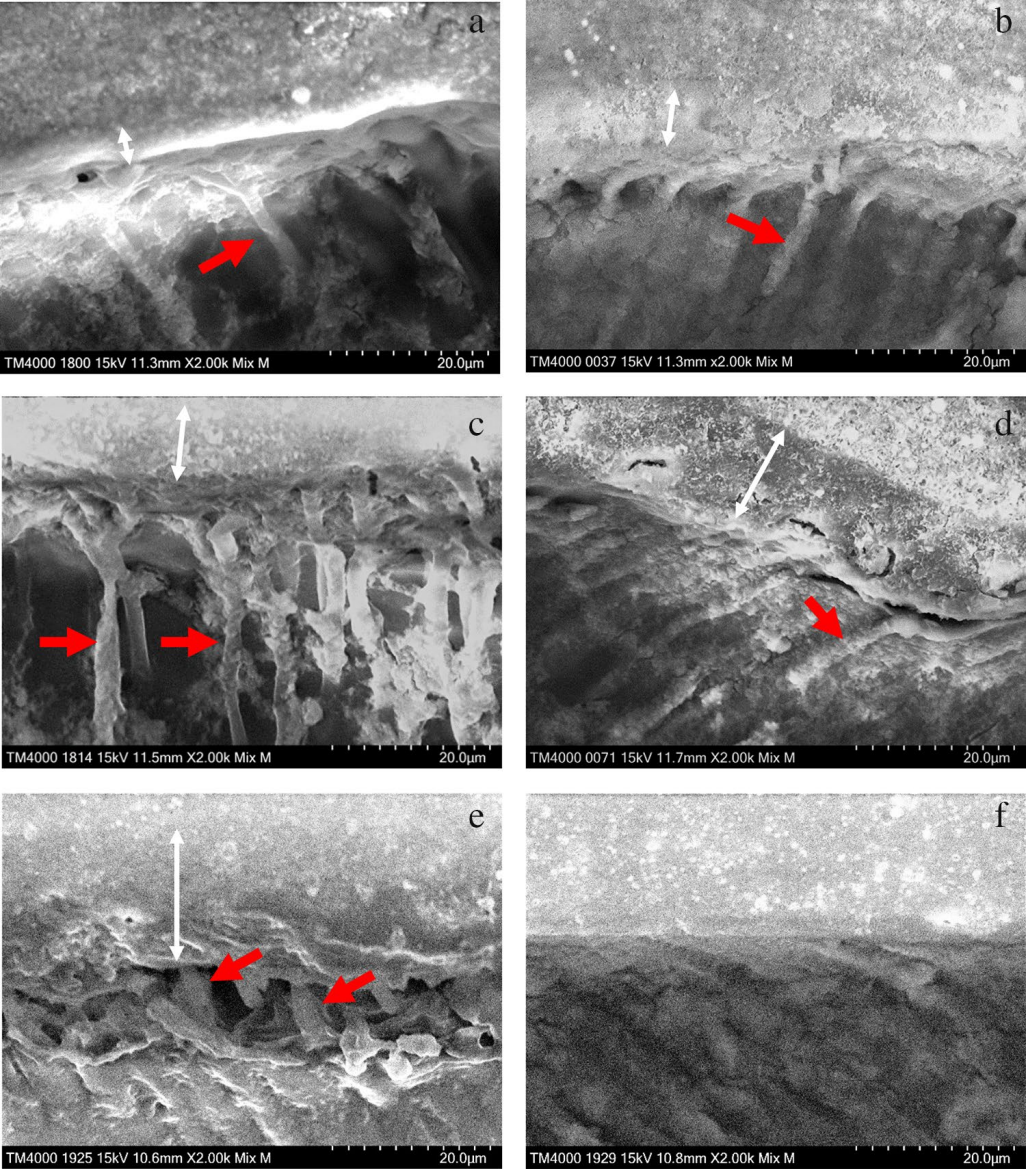
SEM images of the vertical section of the adhesive interface. (**a**–**e**) Specimens in L group (**a**: SI-0, **b**: SI-1, **c**: SI-2, **d**: SI-3, and **e**: SI-4), f: Specimen in G group (SI-0). White arrows: bonding layer, Red arrows: resin tags. Magnification: × 2000. The specimens in the L group show resin tags, whereas the specimen in the G group shows no resin tags. The specimen treated with SI-2 in L group (**c**) shows the numerous resin tags at the adhesive interface.

Representative SEM images of vertical sections of the adhesive specimens are shown in Fig. 9. It was observed that the bonding layer thickness became thicker as the UA viscosity increased in both groups. Further, the uneven bonding layer thickness was greater in the specimens treated with SI-3 and SI-4 than in those with SI-1 and SI-2. On the SEM image of the specimen treated with SI-2 in L group (Fig. 9c), the UA deeply penetrated the dentin tubules to form numerous resin tags at the adhesive interface. Meanwhile, on the SEM image of the specimen treated with SI-0 in G group (Fig. 9f), the thin hybrid layer and few resin tag at the adhesive interface were observed.

## Discussion

Recently, extracted human teeth including deciduous teeth are difficult to collect. Therefore, we used bovine teeth as a substitute for human teeth in this study, because property of bovine tooth is similar to human tooth.

In this study, laser cutting was performed by manual operation on the dentin surfaces flattened with emery paper without using a moving stage. If a moving stage was used for laser cutting, severe damage to the laser tip would have occurred due to an over-contact pressure between the flattened dentin surfaces and laser tip during the moving stage fixed specimen. Therefore, the operator cut the flattened dentin surface using an Er:YAG laser under the appropriate contact pressure of the laser tip to the dentin surface by manual operation. The output power of the laser applied in this study was for healthy dentin, which was higher than that for removing caries dentin^11^. After cutting on the dentin surface with a laser adjusted to the output power for healthy dentin, the dentin surface became clouded. Therefore, the operator could recognize the laser-cut area on the dentin surface by the cloudiness to ensure the laser cutting area.

In a pilot study, we confirmed an uneven roughness of the laser-cut surface when moving the tip vertically or horizontally, whereas even roughness of the laser-cut surface was obtained when moving the tip to draw a circle. Therefore, the operator moved the laser tip on the dentin surface to draw a circle to obtain even roughness of the dentin surface by laser irradiation. A previous study reported that the laser irradiation from an oblique direction for the tooth surface was made more efficiently^33^. However, the laser was irradiated to the dentin surface from a vertical direction in this study because it was difficult to achieve uniform cutting with laser irradiation from an oblique direction.

The effects of laser additional or finishing irradiation under various conditions on the dentin bond strength to RC have been reported^16,23,24^. These reports showed different results that the bond strength decreased when irradiated at low output power^16^, the bond strength improved when irradiated at high output power^24^, and no significant differences were observed among the output powers of laser irradiation^23^. Moreover, the previous study reported that Er:YAG laser pre-treatment with a specific range of energy (50–200 mJ) and frequency (5–20 Hz) improved the shear bond strength between the dentin and the resin in primary teeth^34^. It may be difficult to compare these results because the experimental conditions such as the adhesive system, number of laser pulses, and output power of laser irradiation differed. It would be necessary to examine the effects of laser irradiation conditions on the bond strength of the experimental UA used in this study to the dentin prepared with the Er:YAG laser in future.

BeautyBond Xtream has very low viscosity due to containing no silica, and it is easy to remove water from the applied bond layer by air drying^35^. Therefore, we used BeautyBond Xtream as the matrix of the experimental UA in this study. Kameyama et al. compared the viscosities of all-in-one adhesives such as AQ Bond Plus (Sun Medical, Shiga, Japan), G-Bond (GC, Tokyo, Japan), and Clearfil Tri-S Bond (Kuraray Medical, Osaka, Japan)^19^. It may be difficult to exactly compare the viscosity values between their data and our data because the viscosity measurement conditions differ. However, the results of a simple comparison of the viscosity values among those adhesives used in both studies showed the order of SI-0 < AQ Bond Plus < SI-1 < SI-2 < G-Bond < SI-3 < SI-4 < Clearfil Tri-ES Bond. It may be possible to investigate the effect of viscosity on the dentin bond strength of UAs using products on the market. However, to exclude factors except for the viscosity we prepared and used the four experimental UAs in which the composition was the same but the contents of nanosilica fillers differed.

The L group showed lower dentin bond strength of the experimental adhesives to the dentin than the G group. This result caused the existence of a heat denaturation layer on the dentin surface of the specimens in the L group, which was formed by laser irradiation^36^. The tendency of lower bond strength of adhesives to laser-cut dentin than rotary-cut dentin agrees with the previous studies^12,19–21,28^. These studies reported that the bond strength of a self-etch adhesive to laser-cut dentin was lower than that of rotary-cut dentin because the heat denaturation layer formed on the laser-cut dentin inhibited the adhesive monomer from infiltrating underneath the dentin^37^.

In the G group, the dentin bond strength of specimens treated with SI-3 and SI-4 was significantly lower than that of specimens treated with SI-1, SI-2, and SI-3, suggesting that high viscosity decreased the bond strength of the UA to rotary-cut dentin. These results suggested that the addition of 1 to 2 wt% nanosilica to the adhesive could improve the dentin bond strength of the experimental UA; however, the addition of nanosilica of more than 3 wt% decreased the dentin bond strength of the experimental adhesive. It is well known that the wettability of adhesives to dentin surfaces is dependent on viscosity. Adhesives with higher viscosity have lower wettability to dentin surfaces. A positive correlation between adhesive wettability and bond strength has been reported^38^. The viscosity measurement results of the experimental adhesives clearly showed that the addition of nanosilica increased the adhesive viscosity significantly. Therefore, the null hypothesis that the viscosity of experimentally developed UAs would not influence the bond strength of RC to Er:YAG laser-cut bovine dentin was rejected.

The specimens treated with SI-0, SI-3 or SI-4 in the L group, which exhibited lower bond strength, tended to show mixed failure. The SEM images of the fracture surfaces of the specimen treated with SI-0 in the L group after the µTBS test demonstrated that the partially fractured dentin adhered to the surface of the RC site (Fig. 6), suggesting that the denatured dentin layer was weaker than the intact dentin. On the SEM image of the vertical section, resin tags were observed at the adhesive interface of the specimens treated with SI-0; however, the resin tags were short and fragmented (Fig. 9a). In addition, all specimens treated with SI-3 and SI-4, which were high viscosity UA, showed mixed failure after the µTBS test (Fig. 4), and only a few short resin tags were observed at the resin-dentin interface on the SEM of vertical section (Fig. 9d,e). Whereas, the specimens treated with SI-1 or SI-2 in the L group, which exhibited higher bond strength, tended to show cohesive failure. The high ratio of cohesive failure shown in the higher bond strength groups might be due to the strengthening of the adhesive layer by the addition of nanosilica. On the SEM image of the vertical section, many long resin tags were observed at the adhesive interface of the specimens treated with SI-2 (Fig. 9c). This finding suggests that the high bond strength of SI-2 to laser irradiated dentin may be related to the strong mechanical interlocking generated by numerous long resin tags.

From these results, a much higher viscosity decreased the dentin bond strength of the experimental adhesives, and adjustment of viscosity by adding an appropriate volume of nanosilica seems important for increasing the dentin bond strength of the UAs. It was speculated that resin monomer containing nanosilica in the lower viscosity UAs might penetrate the open dentin tubules on the laser-cut dentin to produce numerous resin tags. As a result, the strengthened adhesive layer including nanosilica generated at the interface between the laser-cut dentin and RC to improve the dentin bond strength. SI-0 without nanosilica (BeautyBond Xtreme, control) may have penetrated the open dentin tubules and produced resin tags, but the tags may have been physically weaker because they did not contain nanosilica. However, the higher viscosity UAs might not be able to penetrate opened dentin tubules enough due to low wettability for dentin surface and form a thick adhesive layer; moreover, the acetone and water in the thick adhesive might not be sufficiently volatilized by air blowing^39^. As a result, an inappropriate bonding layer was formed at the adhesive interface to decrease the dentin bond strength of the higher viscosity UA.

Meanwhile, SI-0 demonstrated high dentin bond strength for the ground dentin as well as SI-1 and SI-2. Although few resin tags were observed at the adhesive interface of the specimen treated with SI-0 (Fig. 9f), which is lower viscosity, the thin adhesive layer stuck to the dentin without a gap, suggesting that a high-quality hybrid layer was generated at the adhesive interface. The UA used in this study can remove the smear layer but cannot be able to open the dentin tubules distinctly due to its inadequate etching capacity (pH = 2.3). Therefore, it was confirmed that the bond strength to ground dentin depends on mechanical adhesion not by the resin tag but by the hybrid layer as reported in a previous study^28^.

In general, the dentin adhesion mechanism of various adhesive systems mainly involves the formation of a hybrid layer, which is generated by an acidic monomer infiltrating the superficial dentin^40,41^. Whereas, a hybrid layer may not be sufficiently generated on the surface of laser-cut dentin because the superficial heat-denatured layer may inhibit the infiltration of the resin monomer^42^. Our results imply the positive effect of adding nanosilica fillers with appropriate contents to the UA on the bond strength to laser-cut dentin. We speculate that numerous long resin tags strengthened by including nanosilica fillers may improve the bond strength to laser-cut dentin.

Within the limitations of this study, it was concluded that the viscosities of the experimental universal adhesives affected their bond strength to both laser-cut and ground dentin, and the experimental universal adhesives deeply penetrated the dentin tubules to form numerous resin tags at the adhesive interface on the laser applied specimens.

## Materials and methods

### Experimental materials

In cooperation with Shofu company, four experimental UAs (SI-1, SI-2, SI-3, and SI-4) were experimentally developed by adding 1, 2, 3, and 4 wt/% nanosilica to BeautyBond Xtreme (Lot #: 200706, Shofu, Kyoto, Japan) respectively. BeautyBond Xtreme, which includes no nanosilica, was used as a control (SI-0). Compositions, contents of nanosilica, and sizes of nanosilica for the experimental UAs were shown in Table 1. The light-cured flowable RC used in this study was BeautyFill Flow Plus X (F00, A1 shade, Shofu).

**Table 1:** Experimental UAs.

UA	Main components	Filler content (wt%)	Filler size (nm)	Lot #	Manufacturer
SI-1	Acetone, Purified water, Bis-GMA, Carboxylic acid monomers, TEGDMA, Phosphate monomer, Silane coupling agents	1	5–50	20201002	Shofu
SI-2		2			
SI-3		3			
SI-4		4			

*UA* Universal adhesive, *Bis-GMA* Bisphenol A-glycidyl methacrylate, *TEGDMA* Triethylenglycol-di-methacrylate.

Extracted bovine mandibular anterior teeth were obtained from slaughterhouse (Niigata Meat Plant, Niigata, Japan). After removing the gingiva and periodontal ligament from the roots of the teeth, they were immediately frozen and stored in a -20 °C freezer until use. When used for experiments, they were disinfected by immersion in a 1% thymol solution for 5 min after thawing. The institution's ethics committee does not conduct ethical review of research use of bovine teeth.

The Er:YAG laser used in this study was Erwin AdvErL EVO (Morita, Kyoto, Japan). The irradiation tip of CS600F, which is suitable for cutting hard tissues, was used for cutting the flattened dentin surface of bovine tooth.

### Measurement of viscosity

The experimental UA viscosity was measured using a B-type viscometer (Digital Viscometer DVL-B, Tokyo KEIKI, Tokyo, Japan) with a coaxial double-cylinder type adapter for a small volume specimen. The viscosities of SI-0 and SI-1 were measured in a beaker entered SI-0 and SI-1 liquid of 20 ml using the L rotor, respectively. The viscosities of SI-2 and SI-3 were measured in a beaker entered SI-2 and SI-3 liquid of 40 ml using the No.1 rotor, respectively. The viscosity of SI-4 was measured in a beaker entered SI-4 liquid of 40 ml using the No.2 rotor.

A thermostatic chamber was used to maintain the temperature of each experimental UA at 25 °C during the viscosity measurement. Because the experimental UA was a non-Newtonian fluid, the rotation speed of the rotors was uniform at 60 rpm. The measurement for each experimental UA was repeated 10 times.

### Preparation of specimens for adhesion test

The labial surfaces of the bovine teeth were ground with #600 emery paper (Carbimet, Buehler Ltd., Lake Bluff, IL, USA) to obtain flattened dentin surfaces using a polishing machine (Lewel specimen polisher, Kasai Co. Ltd., Yokohama, Japan) under water irrigation. The flattened dentin surfaces were shallowly cut by irradiating the Er:YAG laser under the conditions of 100 mJ with 20 pps, which were used in the laser cutting group.

The CS600F tip, which is relatively new in the market since 2015 and possesses easy handling with highly visible properties during tooth cutting, was used. Laser cutting on the flattened dentin surfaces was performed by manual operation. The operator vertically touched the tip to the dentin surfaces and moved the tip as drawing a circle. The laser cutting area were marked on the dentin surface in advance and the marked area were irradiated with the laser using white cloudiness as an indicator.

After each experimental UA was applied to the laser-irradiated dentin surface, the UA was immediately air-blown at low pressure for 3 s to evaporate the solvent, followed by high-pressure air-blowing to remove any redundant residue. In other words, the UA was used in self-etching mode. After photo-curing the bonding layer using the LED light-curing unit (PenCure 2000, Morita) for 5 s at 1000 mW, the flowable RC was placed on the treated dentin surface as a cylindrical shape with a diameter and height of approximately 5 and 1 mm, respectively, following photo-curing for 10 s, which was repeated twice to finally fabricate the cylindrical polymerized RC with approximately 5 and 2 mm in diameter and height. respectively. Five bovine teeth were used for each experimental group.

To investigate the bond strength of each experimental UA to the dentin ground with #600 emery paper without laser irradiation, we also fabricated the adhesive specimens for just ground dentin in the same way. Conveniently, the experimental group assigned laser irradiated dentin and just ground dentin was referred L group and G group, respectively.

### Microtensile bond strength (µTBS) measurement

After the adhesive specimens were stored in distilled water at 37 °C for 24 h storage, the roots were removed using a diamond point (Bur No.105R, ISO size 22; Shofu Inc., Kyoto, Japan), and thereafter, the pulp tissue was removed from their coronal parts. The specimens were longitudinally sectioned into 1-mm thick slabs using a low-speed diamond saw (Isomet, Buehler Inc., Lake Bluff, IL, USA) under water cooling. Two slabs were obtained from each specimen, and each slab was sectioned into two beams using a low-speed diamond saw. The cross-sectional area of the beam was approximately 1 mm^2^. In total, 20 beams were obtained for each experimental group.

The beams were attached to the testing device (Bencor-multi-T, Danville Engineering Inc., San Ramon, CA, USA) with cyanoacrylate (Model Repair Pink, Dentsply-Sankin Inc., Tochigi, Japan), which was placed onto the tabletop material tester (EZ test, Shimadzu Corp., Kyoto, Japan), and then subjected to µTBS test at a 0.5 mm/min crosshead speed (n = 20).

### Failure mode analysis

The specimens after the adhesion test were observed on the fracture surface using a stereomicroscope (SZX7, Olympus, Tokyo, Japan) at × 25 magnification to determine the failure mode. The criteria for judging the respective failure modes are shown in Table 2.

**Table 2:** Criteria for judging the respective failure modes.

Failure mode	Definition of criteria
Adhesive	Exposure of resin/dentin interface is recognized on the fractured surface more than approximately 90%
Cohesive in resin composite	Resin adherence is recognized on the fractured dentin surface more than approximately 90%
Cohesive in dentin	Dentin adherence is recognized on the fractured resin surface more than approximately 90%
Mixed	Other findings are recognized on the fractured surface

### Preparation of specimens for adhesive interface observation

The adhesive specimens were fabricated according to the same manner mentioned above for each experimental group using extracted bovine teeth (n = 2). Each specimen was vertically sectioned for the adhesive interface using Isomet under water cooling. The 40% phosphoric acid gel (Kuraray Noritake Dental, Tokyo, Japan) was applied to the surface of the sections and left for 1 min, then washed with water spray for 5 s and then dried well.

### Scanning electron microscope (SEM) observation

Microstructural observation on fracture surface of representative specimens after μTBS test and vertical section surfaces of the specimens in each experimental group was performed using an environmental SEM (TM4000Plus Miniscope, Hitachi, Tokyo, Japan) at × 100, × 500, and × 2000 magnification.

### Statistical analysis

BellCurve for Excel (Version 3.21, Social Survey Research Information, Tokyo, Japan) was used for statistical analysis. Each data obtained from viscosity measurement and μTBS test was statistically analyzed to detect the significant differences among the experimental UAs. We decided which the parametric or nonparametric test was applied for the statistical analysis after testing the normal distribution and the homogeneity of variance of data using Shapiro–Wilk test and Bartlett test, respectively. The level of significance was set at *p* < 0.05.

### Ethical approval

The contents concerning human and animals were not included in this study.

## Supplementary Information

Supplementary Information.

## Data Availability

All data generated or analysed during this study are included in this published article and its supplementary information files.

## References

[1] Wicht MJ, Haak R, Fritz UB, Noack MJ (2002). Primary preparation of class II cavities with oscillating systems. Am. J. Dent..

[2] Horiguchi S, Yamada T, Inokoshi S, Tagami J (1998). Selective caries removal with air abrasion. Oper. Dent..

[3] Hamama HH, Yiu CKY, Burrow MF, King NM (2013). Chemical, morphological and microhardness changes of dentine after chemomechanical caries removal. Aust. Dent. J..

[4] Liu JF, Lai YL, Shu WY, Lee SY (2006). Acceptance and efficiency of Er:YAG laser for cavity preparation in children. Photomed Laser Surg..

[5] Li T, Zhang XL, Shi H, Ma Z, Lv BJ, Xie M (2019). Er:YAG laser application in caries removal and cavity preparation in children: A meta-analysis. Lasers Med. Sci..

[6] Raucci-Neto W, Chinelatti MA, Palma-Dibb RG (2008). Ablation rate and morphology of superficial and deep dentin irradiated with different Er:YAG laser energy levels. Photomed. Laser Surg..

[7] Colucci V, do Amaral FLB, Pecora JD, Palma-Dibb RG, Corona SAM (2009). Water flow on erbium: Yttrium–aluminum–garnet laser irradiation: Effects on dental tissues. Lasers Med. Sci..

[8] Omae M, Shinnou Y, Tanaka K, Abo T, Nakata T, Suzuki K, Hatsuoka Y, Iwata N, Yoshikawa K, Nishitani Y, Yamamoto K, Yoshiyama M (2009). XPS analysis of the dentin irradiated by Er:YAG laser. Dent. Mater. J..

[9] Raucci-Neto W, Pecora JD, Palma-Dibb RG (2012). Thermal effects and morphological aspects of human dentin surface irradiated with different frequencies of Er:YAG laser. Microsc. Res. Tech..

[10] Raucci-Neto W, dos Santos CR, de Lima FA, Pecora JD, Bachmann L, Palma-Dibb RG (2015). Thermal effects and morphological aspects of varying Er:YAG laser energy on demineralized dentin removal: An in vitro study. Lasers Med. Sci..

[11] Kameyama A, Kato J, Aizawa K, Suemori T, Nakazawa Y, Ogata T, Hirai Y (2008). Tensile bond strength of one-step self-etch adhesives to Er:YAG laser-irradiated and non-irradiated enamel. Dent. Mater. J..

[12] De Munck J, Van Meerbeek B, Yudhira R, Lambrechts P, Vanherle G (2002). Micro-tensile bond strength of two adhesives to Erbium:YAG-lased vs. bur-cut enamel and dentin. Eur. J. Oral Sci..

[13] Firat E, Gurgan S, Gutknecht N (2012). Microtensile bond strength of an etch-and-rinse adhesive to enamel and dentin after Er:YAG laser pretreatment with different pulse durations. Lasers Med. Sci..

[14] Manhaes LA, Oliveira DC, Marques MM, Matos AB (2005). Influence of Er:YAG laser surface treatment and primer application methods on microtensile bond strength self-etching systems. Photomed. Laser Surg..

[15] Memarpour M, Shafiei F, Razmjoei F, Kianimanesh N (2016). Effect of laser preparation on adhesion of a self-adhesive flowable composite resin to primary teeth. Microsc. Res. Tech..

[16] Gisler G, Gutknecht N (2014). The influence of the energy density and other clinical parameters on bond strength of Er:YAG-conditioned dentin compared to conventional dentin adhesion. Lasers Med. Sci..

[17] Nahas P, Zeinoun T, Majzoub Z, Corbani K, Nammour S (2016). The effect of energy densities on the shear bond strength of self-adhering flowable composite to Er:YAG pretreated dentin. BioMed. Res. Int..

[18] Eguro T, Maeda T, Otsuki M, Nishimura Y, Katsuumi I, Tanaka H (2002). Adhesion of Er:YAG laser-irradiated dentin and composite resins: Application of various treatments on irradiated surface. Lasers Surg. Med..

[19] Kameyama A, Aizawa K, Kato J, Hirai Y (2009). Tensile bond strength of single-step self-etch adhesives to Er:YAG laser-irradiated dentin. Photomed. Laser Surg..

[20] Shirani F, Birang R, Malekipur MR, Zeilabi A, Shahmoradi M, Kazemi S, Khazaei S (2012). Adhesion to Er:YAG laser and bur prepared root and crown dentine. Aust. Dent. J..

[21] Saraceni CH, Liberti E, Navarro RS, Cassoni A, Kodama R, Oda M (2013). Er:YAG-laser and sodium hypochlorite influence on bond to dentin. Microsc. Res. Tech..

[22] He ZD, Chen LL, Shimada Y, Tagami J, Ruan SC (2017). Evaluation of sub-surface penetration and bonding durability of self-etching primer systems to Er:YAG laser treated cervical dentin. Dent. Mater. J..

[23] Chen ML, Ding JF, He YJ, Chen Y, Jiang QZ (2015). Effect of pretreatment on Er:YAG laser-irradiated dentin. Lasers Med. Sci..

[24] Aizawa K, Kameyama A, Kato J, Amagai T, Takase Y, Kawada E, Oda Y, Hirai Y (2006). Resin bonding to dentin irradiated by high repetition rate Er:YAG laser. Photomed. Laser Surg..

[25] Kameyama A, Kawada E, Amagai T, Takizawa M, Oda Y, Hirai Y (2002). Effect of HEMA on bonding of Er:YAG laser-irradiated bovine dentine and 4-META/MMA-TBB resin. J. Oral Rehabil..

[26] Kameyama A, Oda Y, Hirai Y, Kawada E, Takizawa M (2001). Resin bonding to Er:YAG laser-irradiated dentin: Combined effects of pre-treatments with citric acid and glutaraldehyde. Eur. J. Oral Sci..

[27] Ayar MK, Erdermir F (2018). Bonding strength of universal adhesives to Er, Cr:YSGG laser-irradiated dentin. Niger. J. Clin. Pract..

[28] Cardoso MV, Neves AD, Mine A, Coutinho E, Van Landuyt K, De Munck J, Van Meerbeek B (2011). Current aspects on bonding effectiveness and stability in adhesive dentistry. Aust. Dent. J..

[29] Memarpour M, Shafiei F, Razmjouei F, Soltani M (2018). Shear bond strength and scanning electron microscopy characteristics of universal adhesive in primary tooth dentin: An in vitro study. Dent. Res. J..

[30] Paryab M, Sharifi S, Kharazifard MJ, Kumarci N (2019). Cavity preparation by laser in primary teeth: Effect of 2 levels of energy output on the shear bond strength of composite restoration to dentin. J. Lasers Med. Sci..

[31] Al-Batayneh OB, Seow WK, Walsh LJ (2014). Assessment of Er:YAG laser for cavity preparation in primary and permanent teeth: A scanning electron microscopy and thermographic study. Pediatr. Dent..

[32] Donmez N, Gungor AS, Karabulut B, Siso SH (2019). Comparison of the micro-tensile bond strengths of four different universal adhesives to caries-affected dentin after ER:YAG laser irradiation. Dent. Mater. J..

[33] De Carvalho RCR, De Freitas PM, Otsuki M, Eduardo CD, Tagami J (2008). Micro-shear bond strength of Er:YAG-laser-treated dentin. Lasers Med. Sci..

[34] Wang JH, Yang K, Zhang BZ, Zhou ZF, Wang ZR, Ge X, Wang LL, Chen YJ, Wang XJ (2020). Effects of Er:YAG laser pre-treatment on dentin structure and bonding strength of primary teeth: An in vitro study. BMC Oral Health.

[35] Saeed NA, Tichy A, Shimada Y (2022). Bonding of universal adhesives to bur-cut dentin: Effect of double application and dentin moisture level. Dent. Mater. J..

[36] Camerlingo C, Lepore M, Gaeta GM, Riccio R, Riccio C, De Rosa A, De Rosa M (2004). Er:YAG laser treatments on dentine surface: Micro-Raman spectroscopy and SEM analysis. J. Dent..

[37] Ceballos L, Toledano M, Osorio R, Tay FR, Marshall GW (2002). Bonding to Er-YAG-laser-treated dentin. J. Dent. Res..

[38] Rosales-Leal JI, Osorio R, Holgado-Terriza JA, Cabrerizo-Vílchez MA, Toledano M (2001). Dentin wetting by four adhesive systems. Dent. Mater..

[39] Fu J, Saikaew P, Kawano S, Carvalho RM, Hannig M, Sano H, Share SD (2017). Effect of air-blowing duration on the bond strength of current one-step adhesives to dentin. Dent. Mater..

[40] Pashley DH, Tay FR, Breschi L, Tjaderhane L, Carvalho RM, Carrilho M, Tezvergil-Mutluay A (2011). State of the art etch-and-rinse adhesives. Dent. Mater..

[41] Van Meerbeek B, Yoshihara K, Yoshida Y, Mine A, De Munck J, Van Landuyt KL (2011). State of the art of self-etch adhesives. Dent. Mater..

[42] Takada M, Shinkai K, Kato C, Suzuki M (2015). Bond strength of composite resin to enamel and dentin prepared with Er, Cr:YSGG laser. Dent. Mater. J..

